# Working in the time of COVID-19: Rehabilitation clinicians’ reflections of working in Gauteng’s public healthcare during the pandemic

**DOI:** 10.4102/ajod.v11i0.889

**Published:** 2022-04-28

**Authors:** Hester M. van Biljon, Lana van Niekerk

**Affiliations:** 1 Department of Occupational Therapy, Faculty of Medicine and Health Sciences, Stellenbosch University, Cape Town, South Africa

**Keywords:** rehabilitation therapists, persons with disability, pandemic, public healthcare users, COVID-19, disorder and confusion, reflective practice, inter-professional communication, disaster, leadership, personal protective equipment, multidisciplinary rehabilitation team

## Abstract

**Background:**

When the coronavirus disease 2019 (COVID-19) pandemic manifested in South Africa, rehabilitation services were seriously affected. The consequences of these were wide-ranging: affecting service users, their families and caregivers, rehabilitation practices and practitioners as well as the integrity and sustainability of rehabilitation systems.

**Objectives:**

This study aimed to explore the nature and consequences of disruption caused by the pandemic, based on the experience of rehabilitation clinicians who were working in public healthcare facilities in Gauteng.

**Methods:**

This was a phenomenology study that used critical reflection method. Trained and experienced in reflecting on barriers and enablers that affect their practices, a multidisciplinary group of rehabilitation clinicians captured their experience of working during the time of COVID-19. Data construction extended over 6 months during 2020. An inductive thematic analysis was performed using Taguette: an open-source qualitative data analysis tool.

**Results:**

The main themes captured the disorder and confusion with its resultant impact on rehabilitation services and those offering these services that came about at the beginning of the pandemic. The importance of teamwork and leadership in rehabilitation also emerged as themes. Other themes related to having to approach work differently, working beyond professional scopes of practice and pandemic fatigue.

**Conclusion:**

The COVID-19 pandemic disrupted the way rehabilitation was being performed, creating an opportunity to reconceptualise, strengthen and improve rehabilitation services offered at public healthcare. The presence of effective leadership with clear communication, dependable multidisciplinary teams and clinicians with robust personal resources were strategies that supported rehabilitation clinicians whilst working during COVID-19.

## Introduction

When disaster, in any form strikes, vulnerable individuals in a community suffer the most (Pedersen 2002). Children, older adults and persons with a disability are identified as vulnerable populations (Murthy & Lakshminarayana 2006) and are frequent users of rehabilitation services. The World Health Organization’s (WHO) stand on rehabilitation (WHO 2020b) is that it is an important part of universal health coverage and a key strategy to ensure healthy lives and promote well-being for all (United Nations 2015). A WHO survey (WHO 2020a) observed that a national disaster, such as the coronavirus disease 2019 (COVID-19), disrupts existing rehabilitation services and has the greatest impact on the most vulnerable populations and the weakest health systems. They acknowledge that whilst the important role of rehabilitation in emergencies is recognised in clinical and humanitarian guidelines, it is rarely considered as part of health system preparedness and early response. The result is that pre-existing limitations in rehabilitation services are magnified, health service delivery is less efficient and people directly affected are at risk of increased impairment and disability.

In March 2020, when the COVID-19 pandemic surfaced in South Africa and a national state of disaster was declared (Ramaphosa 2020), rehabilitation was severely disrupted. Realisation of the magnitude of the challenge faced by rehabilitation practitioners and their patients unfolded and an email (Friday, 17 April, 17:54) sent by the Health Professions Council of South Africa (HPCSA) to all its members, captured the situation:

The COVID-19 pandemic calls upon health practitioners to operate under extraordinary circumstances that fall beyond the current regulatory framework. Healthcare practitioners find themselves on the frontline of the outbreak, selflessly caring for sick and anxious patients, with some practitioners already contracting the virus. The declaration of the state of national disaster in terms of the *Disaster Management Act* requires practitioners registered under the *Health Professions Act* 1974 (the Act) and other health statutory councils to meet challenges which our regulatory framework must accommodate. (HPCSA Team 2020)

The South African healthcare system has been characterised by a stark public–private divide (Keeton 2010) that was created by institutional segregation policies half a century ago. This divide is still prevalent despite governmental efforts to foster universal access to healthcare with attempts that include charters, policies and strategies (Malakoane et al. 2020). South Africa’s public healthcare serves 84% of the population and faces numerous quality improvement challenges (Maphumulo & Bhengu 2019). In addition, it carries the burden of care for communicable diseases such as tuberculosis (TB) and human immunodeficiency virus (HIV), which are particularly high amongst groups with limited socio-economic means (Ataguba, Akazili & McIntyre 2011). During the COVID-19 pandemic the situation prevailed; on 10 April 2020, a month after the pandemic brought about a state of national disaster in South Africa, the number of critical care beds nationally stood at 3318, of which 2140 were in the private sector (Labuschaigne 2020). The rehabilitation services in public healthcare were not exempt from this situation.

Prior to the pandemic there were concerns that rehabilitation services in South Africa were not seen as a healthcare priority, especially so in the resource scarce public healthcare system (Morris et al. 2021). During the initial stages of the pandemic, when lockdown restrictions were most severe, vital disability specific services were not deemed ‘essential’. Concerns prevailed that COVID-19 policies and practices continued to exclude people who require rehabilitation (McKinney, McKinney & Swartz 2020). A survey by the WHO (2020a) confirmed that in many countries rehabilitation services were deliberately suspended by governmental COVID-19 protocols. Such services were further affected by policies limiting or suspending outpatient services and offering only select inpatient services and community-based care. The lasting impact of the COVID-19 pandemic on health services, including rehabilitation, is a source of concern with the likelihood of serious adverse health effects on vulnerable populations, such as children, older persons, people living with chronic conditions or disabilities and minority groups. With the pandemic came a singularly unique opportunity to capture and learn from the experience of rehabilitation practitioners operating under conditions that past and current regulatory frameworks could not anticipate.

Using reflective practice to capture contextual experience is well documented (Knightbridge 2019) and as frequently cited progenitors of reflective practice, Dewey, Schön and McAllister’s work document its value in diverse, multicultural education and healthcare environments (McAllister et al. 2006). Reflection is also a fundamental element in evidence-based practice (Mantzoukas 2008) and widely incorporated into undergraduate healthcare curriculums (Brown, Cosgriff & French 2008). In its simplest form, reflective practice is the ability to reflect on actions and experiences and engage the results of these reflections into processes of learning and professional improvement (Farrell 2018). Evidence generated through reflective practice is contextually grounded and has become an influential concept in healthcare (Fragkos 2016). Within the rehabilitation health sciences there are numerous calls for the promotion of reflective practice. Myezwa et al. (2017) found it improved accountability and learning amongst physiotherapy students and calls for strategies to develop reflective practice. Reid (2009) observed the importance of reflective practice in clinical occupational therapy and called for a culture of mindful practice to be cultivated in the profession. Caty (2014) recorded a considerable need for initiatives involving reflection and reflective practice in speech–language professions.

This research was undertaken to capture the reflections of a group of multi-disciplinary clinicians offering rehabilitation in the South African public healthcare system during a time of extreme disruption. These in action reflections were expected to contain valuable insights that could be used to refine the conceptualisation of rehabilitation and to inform and improve rehabilitation in terms of service, education, leadership and management.

## Research methods and design

### Researcher positionality

Since 2017 the occupational therapy division at Stellenbosch University and the Gauteng Health public sector have had a research liaison that saw four research projects to conclusion. The projects involved multiprofessional teams of rehabilitation clinicians working in various public healthcare facilities. The authors of this article, a post-doctoral fellow and her professorial host, conceptualised and facilitated the research projects. All of which required face-to-face interaction with participating clinicians at the clinical facilities where they worked.

In 2019 the principal author had trained and equipped participants in real-time workshops at their clinical practices for a research project titled: *Reflections on practicing in Gauteng’s rehabilitation services.* Participants in this research, who had given informed consent, were supported, regularly followed up and debriefed on conclusion of the data collection phase in November 2019, by the post-doctoral fellow. March 2020 saw the extension of the same research with a focus change to practicing during the pandemic, involving the same researchers and participants.

### Study design and paradigm

This phenomenology research design saw practicing rehabilitation professionals engaging in a critical reflection method, reflecting on their lived experience of working in the time of COVID-19. Numerous reflective practice tools and models have been developed and are used in healthcare to help professionals reflect on their practices (Fragkos 2016). Most of them incorporate three key elements: to identify, describe or review a situation or action, to explore or examine this from differing angles or viewpoints and to act or plan for action. With knowledge and experience of working in public healthcare rehabilitation practices the researchers decided to choose a simple and easy to use reflection process, as found in Kolb’s experiential learning style theory (Kolb 1984). Kolbs’ theory was used in the training of physiotherapy – (Aldegether 2017), occupational therapy – and speech and hearing students (Brown et al. 2008). His reflective cycle of concrete experience, reflective observation, abstract conceptualising and active planning/learning (McLeod 2017) and has also been used in clinical rehabilitation practice (Knightbridge 2019).

### Setting

In 2019, physiotherapists, assistant physiotherapists, occupational therapists, occupational therapy technicians and assistants, podiatrists, speech and hearing therapists, speech therapists and audiologists, working in public healthcare facilities were recruited and trained to reflect on their practices. The aim of the research was to identify barriers and enablers that affected their rehabilitation practices. In 2020, when the COVID-19 pandemic became evident in South Africa, a participant suggested continuation of reflective practice with a focus on working during the pandemic. Other participants confirmed the need, to capture and share their unique experiences. Gauteng Health head office and ethical clearance was received for the amended and extended research project. As a result of pandemic restrictions all contact between researchers and participants was in the form of electronic and telephonic communication.

### Research population and sampling strategy

A convenience sample was obtained by inviting all participants involved in the 2019 research to participate. All participants employed by Gauteng Health public sector, underwent training in reflection and had experience of professional reflection. Recruitment was performed via e-mail communication containing information on the amended and extended research project, a consent form and an invitation for participation. In cases of no response to the initial e-mail, follow-up communication was performed to ensure that participants received the information. Clinicians who volunteered to take part in the research signed consent forms. The two researchers, authors of this article, were employed by Stellenbosch University and had no association with Gauteng public healthcare.

### Data collection, processing and analysis

Participating clinicians were assigned personal research codes, provided with an electronic reflection guide (e.g. refer to Appendix 1) and instructions on submission of their reflections to the researcher. Participants were asked to reflect once a week and to capture these electronically. Reflections could comprise once-off experiences or be further expanded and developed in subsequent reflections. A target of at least 10 reflections was set. The first author developed the reflection guide, adapting it from the 2019 research that was extended. The researchers had prior experience in action research (Coetzee et al. 2011; Van Biljon 2016) and the use of professional reflection in public healthcare research (Van Biljon, Casteleijn & Du Toit 2015).

Data collection comprised completion of demographic details, reflection in practice, which was guided by a reflection guide (e.g. see Appendix 1) and field notes taken by the first author during once monthly email and telephonic follow-up and support conversations with participants. Participating clinicians reflected over 6 months from 14 April 2020 to 30 October 2020 and captured 130 reflections. During this time, they were followed up and supported by the first author through electronic communication.

The two authors were the data coders and themes were not proposed in advance but derived from the data. Demographic data were summarised using descriptive analysis. Reflections and field notes were imported into Taguette, a free open-source text tagging tool for qualitative data analysis, where they were analysed using inductive thematic content analysis. Creswell’s fully integrated analysis and integration approach was used (Cresswell 2013).

### Trustworthiness

Credibility was enhanced by using a reflection guide that was used in and refined in a similar research project study with the same participants. A clear audit trail and transparency was maintained during all phases of the research. Confirmability was obtained through individualised, prolonged engagement andcoding of the data. Three forms of data triangulation were done; time and space triangulation involved collection of data at different intervals and in several settings whilst person triangulation was done by collecting data from various participants. Transferability of the data should be judged with caution and consideration of the South African context. South Africa is an upper-middle-income economy with limited rehabilitation services available to uninsured public healthcare service users. However, the disrupting impact of the COVID-19 pandemic and subsequent restrictions experienced might be similar in many countries across the globe.

### Ethical considerations

Ethical considerations included the risks associated with professional reflections. Confidentiality and autonomy of participants was a priority with only the first author having access to identifiable information that could link reflections to participants; personal research numbers were used on reflection forms to ensure cohort anonymity. Participants, who signed consent forms, were informed of their right to decline participation at any time without consequences, and given the opportunity to ask questions, discuss and contemplate their participation. Compliance with the principles of the Declaration of Helsinki was prioritised throughout the research. Stellenbosch University’s Human Research Ethics Committee (HREC) provided ethical approval (Ref No N18/01/113) for the amendment and extension of the research. Gauteng Healthcare’s Research Committee approved the research with ref no: DRC Ref 2018-03-008, and the research was registered on the South African National Health Research Database (Ref. No GP201802022). Continuing Professional Development (CPD) points were awarded to participants who did not receive any other form of remuneration.

## Findings

Of the 75 participants who were invited, 19% (*n* = 14) volunteered, were informed regarding the research, signed consent forms and provided demographic information. On conclusion of the data collection phase, eight rehabilitation clinicians (57% compliance) submitted reflections. Reasons for non-completion provided by the six participants who did not complete data collection, were inertia and feeling overwhelmed.

Demographic details of the eight clinicians who completed data collection show a gender distribution of one male and seven females, the average age was 40 years (Range 26 – 58) and all were South African citizens. Reported home languages were English (*n* = 4), Afrikaans (*n* = 2), IsiXhosa (*n* = 1) and Sepedi (*n* = 1). Professions represented were occupational therapy (*n* = 5), physiotherapy (*n* = 1), podiatry (*n* = 1), speech and audiology (*n* = 1). All had tertiary-level qualifications with an average of 18 years’ clinical experience (range 2–40). All participants worked in tertiary healthcare facilities: one performed only management functions, two combined management with clinical responsibilities, the rest were clinicians.

For the quotes, the three unique identifiers were used in brackets: (1) an indicator of the origin of the quote e.g., date of the reflection or field notes which were undated summaries or notes made by participants or researchers; (2) participant code and (3), occupation – using the HPCSA identifier of such.

Field notes by the first author, disclosed reasons why participants were working where they were and to what extent their work had changed. The most prominent reason for working in public healthcare was related to the type of service users they were seeing:

‘[… *H*]ere I can be of the most benefit to the community and … to make a difference in the lives of vulnerable people.’ (Field notes, A1, OT)

Public healthcare also offered them the opportunity to work in large multidisciplinary teams, learning- and experience opportunities, the security of a steady income and having fixed working hours were also mentioned.

On conclusion of the research, they were asked if their work had changed since the pandemic and 60% (*n* = 5) reported changes to the type and volume of work they did. Those who felt their work had only changed partially held management posts. The changes reported were reduced patient loads and having to assist with pandemic-related tasks such as working at screening stations. At some institutions’ rehabilitation personnel were told to work reduced hours and a clinician reported:

‘So many people have lost their jobs because of COVID-19 and I started feeling really guilty that I am only working 2 days a week and still getting my full salary.’ (Field notes, BA1, STA)

From the clinicians’ reflections three dominant and several minor themes emerged, namely *disorder and confusion, impact on rehabilitation services and impact on personal well-being* of rehabilitation practitioners. In addition, *mitigating factors* and evidence of *pandemic fatigue* came to light.

### Theme 1: Disorder and confusion

The disruption and uncertainty brought by the COVID-19 pandemic was pervading every aspect of participants’ professional and personal lives. The sense of disorder and confusion that resulted was evident in participants’ reflections, underlying most of what they were experiencing on a day-to-day basis. The reflection by one of the manager participants aptly captured the emotions experienced during the first months of the pandemic:

‘Anxiety and fear were the reigning emotions. Staff were convinced they had all had some contact with patient. The irrational thoughts and messages between staff caused such mayhem. Made us aware how fully unprepared we were to deal with the inevitable. It took calm and at some points an authoritarian response to have people actually listen through their fears and understand protocols and procedures. I myself had a sense of hopelessness come over me at some point and needed to take some time just to re-centre and face the onslaught of the fear mongering.’ (April 2020, RM2, PT)

There were reports of mandatory personal protection equipment (PPE) running out, frustration and anger about corruption and the misuse of resources. Reflections captured participants’ fear that the systems for implementation of COVID-19 regulations caused confusion and were ineffective:

‘Entering the hospital continued to be an early morning frustration as some days I was told to move my parking, other days I was told to wait in a queue and most days the hermometer did not work!’ (June 2020, AMH1, OT)

A participant with management duties reported:

‘Staff are anxious and unsure of what they are exposed to, what their role is, how safe they are. I spend a lot of time to calm them down ….’ (March 2020, RM4, OT)

Practicing outside the scope of professions’ normal practice required additional training and caused anxiety. Rehabilitation practitioners were required to assist in the swabbing tents, where screening and testing for COVID-19 was done. In the reflection below a clinician shares the challenges associated with learning to take a swab:

‘I was shaking so much I missed the nostril and poked them in the face.’ (May 2020, BA1, STA)

### Theme 2: Impact on rehabilitation services

The negative impact of the pandemic and restrictions imposed to deal with it affected participants’ ability to render rehabilitation services. Certain types of rehabilitation interventions such as vocational rehabilitation, group sessions, family meetings and caregiver training were restricted or were not possible to offer. Clinical student supervision was severely restricted. Interventions that rely strongly on verbal communication were especially affected:

‘I have found that it is incredibly difficult to render quality speech therapy with full PPE on. The nature of communication therapy involves a lot of talking or the patient needing to see your mouth. This isn’t possible with full PPE. The patient is also wearing a surgical mask, which you remove for parts of your assessment but needs to be worn for others making it really difficult to hear the patient.’ (July 2020, BA1, STA)

Concerns related to patients being discharged prematurely, outpatient clinics closing, outpatients arriving for rehabilitation appointments and being refused entrance to the hospital were recorded. Patients were also reported to be *scared of coming to hospital,* which further affected follow up and raised concerns about compliance. A participant reported:

‘I deal mostly with diabetics’ feet. If my patients cannot see me there is going to be lots of complications.’ (May 2020, JH5, CH)

### Theme 3: Impact on personal well-being

The magnitude of disruption brought by the pandemic had a direct and enduring personal impact on participants. Fears, confusion and concerns for the safety of patients, family and friends and participants themselves were a strongly evident theme underlying participants’ reflection. None of the participants had tested positive for COVID-19 during this research period, with three of them responding *not yet* to the question. There was a tone of resignation that showed during a supportive follow-up conversation:

‘[…*I*]t is not if we are going to get it but when.’ (Field notes, A1, OT)

They were anxious about contracting the virus and apprehensive about the possibility of them infecting their patients or families:

‘Thankfully, my results were received this evening and they were negative. First thing I did was hug my kids.’ (May 2020, RM2, PT).

There were also concerns about family members who had contracted COVID-19 and regrets of not being able to attend funerals, religious and cultural ceremonies.

### Minor themes: Mitigating factors, pandemic fatigue and the effect of reflection practice

Several mitigating factors came to light that supported and strengthened rehabilitation clinicians, as a group, but also individually.

The importance of the rehabilitation team and leadership was illustrated with both positive and a negative reflection. Absent or inconsistent leadership led to poor communication and contributed to the confusion and anxiety as shown in the reflection by one of the clinicians:

‘Lockdown is announced. This was very uncertain. We were not given any indication from our managers/supervisors whether we are still working, frequency of work, etc. Several colleagues asked on our departmental WhatsApp group and received the answer that no news means nothing changes. Whilst I understand that our HOD had not received anything from her boss, etc., it still leaves an unsettling feeling.’ (March 2020, TH2, OT)

Conversely, reflections captured just how difficult it was to be in a position of leadership in the absence of protocols and in teams that required leadership on a broader scope of issues than was usually the case. One of the manager participants experienced the situation as follows:

‘I feel that compassion fatigue is setting in and want to scream I DON’T KNOW more than ever before. Some staff seems to be so immobilised by fear and is unable to take the smallest of decisions.’ (March 2020, RM4, OT)

Communication within and between professional teams was shown to contribute positively or negatively to services and the well-being of clinicians. The importance of a supportive team was also observed:

‘Most of my staff have recovered and are back from quarantine. Glad to see our work family reasonably whole again. The positives I have seen is staff coming together to support each other emotionally. Sending words of encouragement on groups and simple thank you’s instead of just being bombarded by bad news.’ (July 2020, RM2, PT)

The personal robustness of individual clinicians also came to light. Rehabilitation clinicians observed their need of *wanting to help*, but not knowing what to do, which increased their anxiety and resulted in feeling overwhelmed and inertia. They felt *guilty* because their workloads were affected and concerned about their patients not having access to rehabilitation. There were also reflections that showed rehabilitation clinicians creativeness, willingness to move beyond their scope of practice and rising to the demand of the occasion before them. Reflections from a clinician who initially reported anxiety and confusion when working beyond her scope of practice in the swabbing tests illustrates this:

‘Being a part of our “swabbing tent initiative” was a powerful experience for me. It made me feel like I was contributing to our hospital’s response to the pandemic and being useful beyond the realms of my professional scope. I am so glad I was a part of the initiative and I am proud of all that we have achieved. Allieds are the sticky, delicious peanut butter and jam holding the public health sector sandwich together, and we proved that yet again with our willingness to go above and beyond.’ (May 2020, BA1, STA)

The success of supportive initiatives such as employee assistance programmes (EAPs) and employee health and wellness programmes were also reported. Gratitude was expressed towards management members who had circulated rehabilitation-specific pandemic information, negotiated for and secured PPE relevant to rehabilitation and organised training sessions for adapted programmes.

Reflections captured towards the end of the research, which was after the first COVID-19 wave and before the second in South Africa, showed pandemic fatigue:

‘I am OVER corona. I am over social distancing. I am over wearing masks. I am over feeling like there are so many people out there who need help but are unable to get it.’

‘I feel [*countries that experienced the pandemic before South Africa and international organisations*] lied to us. I feel millions of Rands have been wasted for nothing. I feel this virus is no worse that TB, which kills more people in South Africa every single year than this virus ever will. I feel the majority of South Africa is really struggling to support their families and those that were already vulnerable have been pushed even deeper into their pit of despair. The knock-on effect, financially, will take years to recover if it ever does. I don’t know if it was really worth it.’ (September 2020, TH2, OT)

The lifting of restrictions allowed for continuation of some familiar rehabilitation initiatives; this went a long way to reduce pandemic fatigue:

‘Heritage Day, physio week, OT week and deaf awareness campaign all provided some much-needed distraction, respite from the COVID-19 onslaught and a glimpse into some form of normalcy. We have had to however keep reminding each other not to become complacent. Having attended some external meeting this month was probably one of the most healing interventions for my heart and soul. I didn’t realise how much I had really missed those interactions. I thrive on interacting with others especially those I regard as mentors and finally seeing these important people in my life was a blessing.’ (September 2020, RM2, PT)

Field notes taken during the conclusion of the research revealed that several participants experienced the systematic capturing of their professional reflections to be a positive experience. It helped them cope with anxiety and uncertainty. One participant referred to the experience as *cathartic*. Another participant captured her views in an email as follows:

‘Just finalising my last entry so I can submit. As much as I started this for the CPD points, reflecting on my experiences over the past few months have put things into some perspective. I was able to share some of these thoughts with colleagues and we could guide and pull each other up when we most needed it. Knowing that I was not alone in this and others were feeling the same makes me feel less isolated.’ (Field notes, AMH1, OT)

## Discussion

Disorder and confusion, disruption of rehabilitation and the strain under which rehabilitation clinicians were, showed how the role of rehabilitation and its service to public healthcare users was challenged. The need to strengthen and improve rehabilitation services offered at public healthcare facilities was demonstrated. Different leadership experiences were reflected on showing how the presence of effective leadership with clear communication, dependable multidisciplinary teams and robust personal resources were strategies that supported rehabilitation clinicians whilst working during COVID-19. Similarly, the positive impact of well-informed clinicians implementing COVID-19 related policies in their practices illustrated the power of good leadership to yield positive outcomes, providing containment that made clinicians feel safe and cared for, reasserting their commitment to their work.

The COVID-19 pandemic has offered an opportunity to learn from the experiences of rehabilitation clinicians, thus producing practice-based evidence that could inform policies or strategies to raise the level of preparedness for future disruptive events. In addition, the consequences of discontinued, restricted or disrupted rehabilitation led to a reappraisal of rehabilitation as an essential service. The findings further highlighted professional competencies, often considered ‘soft skills’, to be paramount in managing heightened demand for containment and mental health concerns of health professionals and their patients alike.

### Strengths and limitation

Qualitative research does not necessarily require large samples to yield good quality findings. However, having only one participant for most of the professions completing data collection is considered a limitation. Conversely, none of the themes that emerged pertained to profession-specific issues, thus reducing the impact of this limitation. The prospective nature and eliciting practice-based reflections was a strength of the research, especially because reflections extended over 6 months. As such the way in which participants were affected by the progression of the pandemic was shown.

### Implications and recommendations

The COVID-19 pandemic has created an opportunity to reconceptualise, strengthen and improve rehabilitation services in public healthcare.

Effective leadership, clear communication, strong multidisciplinary teams and clinicians with robust personal resources, strengthened rehabilitation services offered in public healthcare.

## Conclusion

In Africa, the need to address epidemics, plagues, pandemics and other large-scale threats to health is not an uncommon event. The focus of intervention and planning for such event is often on the role of medical doctors, nurses and community health workers. Evidence that could inform the role of rehabilitation healthcare workers would be of value to public healthcare users, educators, policymakers and professional bodies. Interviewing 101 leading thinkers from a variety of fields, on what the world might look like after the corona virus, Najam concludes that the COVID-19 pandemic will leave nothing unchanged and that it brought about an opportunity for fundamental restructuring (Najam 2021).

For rehabilitation, the pandemic has shown the negative consequences of diverted focus of policymakers and resources, leaving the service users of rehabilitation side-lined. Without strong rehabilitation-focussed leadership and clear policies, rehabilitation services will once again be left floundering with practitioners taking strain. An important lesson for rehabilitation practitioners was the need to move beyond an institution-based silo-practice mindset. Instead, rehabilitation professions should work together to develop collective strategies to ensure the availability of their services. Advocacy for continuation of rehabilitation as an essential service should be a priority of professional associations in preparation for future disasters requiring quick action and clear protocols. Further in-depth exploration involving larger groups of rehabilitation professions from a greater variety of clinical settings, could be valuable to inform restructuring and suggestions emanating from this research.
